# Student Perceptions in Measuring Teaching Behavior Across Six Countries: A Multi-Group Confirmatory Factor Analysis Approach to Measurement Invariance

**DOI:** 10.3389/fpsyg.2020.00273

**Published:** 2020-02-21

**Authors:** Stéfanie André, Ridwan Maulana, Michelle Helms-Lorenz, Sibel Telli, Seyeoung Chun, Carmen-María Fernández-García, Thelma de Jager, Yulia Irnidayanti, Mercedes Inda-Caro, Okhwa Lee, Rien Safrina, Thys Coetzee, Meae Jeon

**Affiliations:** ^1^Department of Public Administration, Radboud University, Nijmegen, Netherlands; ^2^Department of Teacher Education, University of Groningen, Groningen, Netherlands; ^3^Department of Mathematics and Science Education, Çanakkale Onsekiz Mart University, Çanakkale, Turkey; ^4^Department of Education, Chungnam National University, Daejeon, South Korea; ^5^Department of Educational Sciences, University of Oviedo, Oviedo, Spain; ^6^Department Educational Foundation, Tshwane University of Technology, Pretoria, South Africa; ^7^Department of Biology and Biology Education, State University of Jakarta, East Jakarta, Indonesia; ^8^Department of Education, Chungbuk National University, Cheongju, South Korea; ^9^Department of Music Education, State University of Jakarta, East Jakarta, Indonesia

**Keywords:** cross-country comparison, measurement invariance, secondary education, student perceptions, teaching behavior

## Abstract

The purpose of this study is to examine measurement invariance of scoring of teaching behavior, as perceived by students, across six cultural contexts (Netherlands, Spain, Turkey, South Africa, South Korea, and Indonesia). It also aims to compare perceived teaching behavior across the six countries based on a uniform student measure. Results from multi-group confirmatory factor analyses (MGCFA) showed perceived teaching behavior in the six countries to be adequately invariant. Perceived teaching behavior was the highest in South Korea and the lowest in Indonesia. The findings provide new insights into the relevance and differences of teaching behavior across cultural contexts.

## Introduction

Student perceptions are a powerful tool for measuring effective teaching practices in the classroom (den Brok et al., 2006; König and Pflanzl, 2016). However, most studies on perceived effective teaching are limited to one particular setting/country (e.g., Opdenakker et al., 2012; Fernández-García et al., 2019). Although single-country studies can give valuable insights on effective teaching in general, the transferability of the findings to other country contexts is limited due to the lacking clarity regarding the relevance of the constructs in other diverse contexts. Furthermore, existing research from various cultural settings typically use different measures to assess teaching practices. Different measures may assess different constructs. Additionally, single measures can vary significantly with regard to applicability in different educational and national contexts due to differential external validity (Ko and Sammons, 2013). To justify core comparisons across countries, construct and measurement equivalence invariance should be investigated.

Comparing student perceptions of effective teaching across countries is valuable for several reasons. First, it contributes to the increment of knowledge regarding effective teaching behavior across national contexts from the lens of students. Similarities and differences in perceived teaching practices across various countries could be detected and compared (Adamson, 2012). Second, it offers a platform for international benchmarking based on student perceptions. Third, it provides valuable information high quality teacher behavior across various national contexts. Fourth, it provides information for schools on how to improve criteria for (self-) evaluation. Additionally, it contributes to proposals for policy makers in the form of perceived best-practices across countries (Adamson, 2012).

However, comparison across countries is meaningful only if there is sufficient evidence that the same construct of teaching quality is being measured. This psychometric property, also known as *measurement invariance* (Meredith, 1993), should be established before interpreting differences between countries as actual differences. Although scale scores invariance in international large scale achievement tests such as the Programme for International Student Assessment (PISA) and the Trends in International Mathematics and Science Study (TIMSS) has received substantial attention in academic research (Rutkowski and Svetina, 2014), the application of invariance testing in non-achievement surveys is relatively novel. To date, the knowledge about measurement invariance of student perceptions of effective teaching across countries is still largely lacking in the international literature. Research on student perceptions of teachers’ instructional quality based on the PISA 2012 data from the United States, Australia, and Canada shows that effective instructional construct is invariant across the three English speaking countries (Scherer et al., 2016). However, it remains unclear whether the invariant construct of teaching behavior will be evident when data from non-Western, and developing countries are included.

Researchers addressing measurement invariance have so far focused on using classroom observations to measure effective teaching across countries (e.g., van de Grift et al., 2017) and across groups within a country (e.g. Jansen et al., 2013; Fernández-García et al., 2019). Consequently, the direct comparison of effective teaching based on student ratings cannot yet validly be made when measurement invariance is not established beforehand.

The current study therefore aims to examine measurement invariance of student perceptions for measuring effective teaching across six countries: Netherlands, Indonesia, South Korea, South Africa, Spain, and Turkey. In these countries, effective teaching is studied from the perspective of observable teaching behavior based on teaching and teacher effectiveness frameworks. Furthermore, we aim to compare perceived teaching behavior across countries based on a comparable student perceptions measure. This measure was initially developed in Netherlands and has been proven to be useful for measuring perceived effective teaching in research and teacher professional development contexts (Maulana et al., 2016). As noted by Markus (2016), the world does not consist of only WEIRD (Western, Educated, Industrialized, Rich, Democratic) countries, which strengthens the assumption that perceptions about a particular construct may not be shared outside a particular cultural context. It is therefore imperative that a particular construct (i.e., effective teaching) developed in a specific context be tested in other cultural settings.

In the study, multi-group confirmatory factor analyses (MGCFA) were employed using a structural equation model (SEM) framework used to study perceived effective teaching practices across countries. More specifically, the main aim is to answer the following research questions:

1. To what extent is there evidence of an invariant internal structure regarding student perceptions of teaching behavior across countries?
2. How does perceived teaching behavior differ across countries?
  2.1 Which countries were rated higher and on which teaching domains?
  2.2 What is the most complex teaching behavior domain based on student perceptions?

### Theoretical Framework

#### Teaching Behavior

Research on teaching provides strong evidence regarding the highly important role of teaching behavior for student learning outcomes (Seidel and Shavelson, 2007; Hattie, 2009). Hence, the construct has received much attention internationally. Teaching behavior is viewed as complex and multidimensional in nature (Shuell, 1996). Ko and Sammons (2013) summarized existing definitions of teaching behavior. In the present study, we use the operative definition of teaching behavior focusing on the effectiveness of observable behaviors as seen in the classroom in a regular lesson. Effective teaching behavior is defined as teachers’ behavior that has been shown to have an impact on student outcomes (i.e., motivation, engagement, achievement) (van de Grift, 2007). According to reviews of research on the relationships between the basic characteristics of teaching and the students’ academic outcomes, there are several observable teaching behavior components that are closely connected to the effectiveness of teaching. These components include creating a safe and stimulating *learning climate*, exhibiting efficient *classroom management*, displaying *clear instruction*, *activating teaching*, employing *differentiation*, and implementing *teaching learning strategies*. The conceptualizations of teaching behavior domains as described by van de Grift (2007) largely coincide with those of domains described in other widely used teaching behavior frameworks such as the Framework for Teaching of Danielson (2013) and Classroom Assessment Scoring System (CLASS) of Pianta and Hamre (2009).

#### Student Perceptions of Teaching Behavior

For feedback and accountability purposes, determining a valid and reliable measure of effective teaching is important (Timperley et al., 2007). Effective teaching behavior, however, is a complex concept comprising multiple and sequential components. Scheerens et al. (2007) distinguished sequential components of effective teaching behavior into pro-active (preparation before teaching is conducted), interactive (execution of teaching) and retro-active (evaluation of the executed teaching) components. van de Grift (2007) distinguished the component of effective teaching behavior into observable and non-observable elements. Particularly, quantitative measurements have been applied to measure the interactive and observable component of effective teaching behavior.

In general, there are three common tools for measuring teaching behavior: classroom observations, student surveys, and teacher surveys (Lawrenz et al., 2003). The three tools have strengths as well as weaknesses in measuring teaching behavior. Classroom observations have been used predominantly to measure teaching behavior, particularly in primary education (Goe et al., 2008). Classroom observations are viewed as the most objective method of measuring teaching practices (Worthe et al., 1997). This method is recognized as an important procedure in the teacher training process (Lasagabaster and Sierra, 2011). Classroom observations allow judgments about what is happening in the classroom, and these judgments are assumed to be “free” from the influence of students and teachers (Lawrenz et al., 2003). Nevertheless, the presence of observers can influence teachers’ behavior (de Jong and Westerhof, 2001), which can compromise the measurement of typical teaching behavior. Moreover, classroom observations are recognized as very demanding and time consuming because observers should be trained intensively and lessons should be observed multiple times to obtain objective and accurate measures of teaching behavior (Hill et al., 2012; van der Lans et al., 2015).

Student and teacher surveys are known to be cost-effective, less demanding, and less time-consuming for measuring teaching behavior (Goe et al., 2008; Fraser, 2012). Information gathered from surveys is based on teachers’ and students’ classroom experiences over a relatively long period of time, which strengthens the usefulness of surveys for measuring teaching behavior (Ferguson and Danielson, 2015). In practice, it is often difficult to obtain sufficient variations in teacher reported teaching behavior, which has consequences on the flexibility of applying certain statistical analyses. Teacher perceptions of own teaching behavior was also found to be less predictive of student outcomes compared to that of student perceptions (Scantlebury et al., 2001).

Student surveys, more specifically, can be aggregated to the class level in order to obtain information that is comparable to classroom observations (de Jong and Westerhof, 2001). The use of multiple student raters in a class to evaluate teaching behavior reduces rater bias perceptions (Kyriakides, 2005; Goe et al., 2008). Students’ perceptions of classroom processes may actually be more important than what outsiders would observe since student perceptions steer their own learning behavior, based on their own insights. Indeed, studies indicate that student perceptions are mostly more predictive of student outcomes than external observations (de Jong and Westerhof, 2001; Seidel and Shavelson, 2007) and teacher perceptions (Scantlebury et al., 2001). Research also indicates that student perceptions are significantly related to teacher perceptions of their teaching behavior and that the construct structure of teaching behavior based on student and teacher perceptions is similar (Kunter et al., 2008).

Like other measures, using student perceptions for measuring teaching is also subject to criticisms. The critic is mainly related to student ratings as being non-objective because their perceptions are influenced by various factors including their interpersonal closeness with their teachers, interest in the subject taught by their teachers, expectations about their grades, and student age (Peterson, 2000; Richardson, 2005; Benton and Cashin, 2012). Nevertheless, student perceptions can provide valid and trustworthy evaluations of teaching practices (Marsh, 2007). The reliable and valid use of student perceptions is evident for a wide range of educational levels including primary school, middle school, and high school (Peterson et al., 2000). This evidence is extended across various English-speaking countries including Australia, Canada, and the United States (Scherer et al., 2016). In addition, biases derived from student ratings are generally small (Richardson, 2005; Marsh, 2007; Benton and Cashin, 2012). Studies indicate that students are able to discriminate between effective teaching constructs even at the primary school level (van der Scheer et al., 2019). Also, there is evidence that student and teacher perceptions about teachers’ teaching practices are sufficiently invariant, which suggest that both students and teachers interpret the construct of effective teaching behavior similarly (Krammer et al., 2019). Therefore, student evaluation of teaching has been one of the most widely used indicators of teacher effectiveness and educational quality (Scherer et al., 2016).

#### Complexity Level of Teaching Behavior

Teaching behavior is a complex act in a complex environment (Shuell, 1996). It occurs simultaneously but also concerns acts taking place at different duration and time scales (Boshuizen, 2016). To understand the complexity of teaching, the theory of teacher concerns (Fuller, 1969) has been useful in explaining general progressive changes of concerns. According to this theory, teacher concerns follow a stage-like model, starting with concerns with the s*elf*, moving to concerns with the *tasks*, and finally turning to concerns with *impacts* on students (Conway and Clark, 2003).

Grounded on Fuller’s theory of concerns, research on student perceptions of Dutch pre-service teachers’ teaching behavior indicates that perceived teaching behavior follows a stage-like model with increasing complexities (Maulana et al., 2015b). Findings show that, in general, teaching behavior domains related to learning climates and classroom management are positioned in the lower complexity level (concerns with the self), clarity of instruction and activating teaching in the medium complexity level (concern with the task), and differentiation and teaching learning strategies in the higher complexity level (concern with the impact on students).

Findings from classroom observation studies in various international contexts using this and similar teaching behavior frameworks show similar patterns of teaching behavior complexity levels, with differentiation appearing to be the most difficult skill to display in classroom teaching in Netherlands (e.g., van de Grift et al., 2014), Germany (Pietsch, 2010). The complexity of differentiation is well-documented in the literature of teaching (van Geel et al., 2019).

#### Perceived Teaching Behavior Across Countries

Despite the popularity of using student perceptions for measuring effective teaching in their classes, particularly in the context of international large scale studies such as PISA and TALIS, research on student perceptions of teaching behavior across countries is scarce. Hence, evidence of measurement invariance about perceived teaching behavior across cultural contexts is limited. A limited number of studies on measurement invariance of non-achievement constructs exist, which paves the way for further studies on cross-country comparisons in perceived teaching practices.

Using the PISA 2012 data, Scherer et al. (2016) investigated the measurement invariance of student perceptions of teachers’ instructional practices (i.e., teacher support, cognitive activation, classroom management) in Australia, Canada, and the United States using the continuous multi-group confirmatory factor analyses. They found that the constructs were adequately equivalent in the three English-Speaking countries. Furthermore, Desa (2014) studied the measurement invariance of teacher perceptions of effective instructional teaching behavior using TALIS 2008 data and found that the teaching behavior constructs (i.e., teacher-student relationship, classroom disciplinary climate, self-efficacy) were sufficiently equivalent across 23 countries, especially from categorical multi-group confirmatory factory analyses.

In summary, a limited number of studies on perceived teaching practices across countries suggest that measurement invariance of non-achievement constructs can be established. This makes it possible to investigate the perceptions of teaching practices across countries. However, the existing studies also suggest that results of measurement invariance testing may depend on the teaching quality constructs being studied and the statistical approaches employed to test for score comparability.

### Contexts of the Current Study

#### Netherlands

The Dutch educational system is highly tracked, students are separated by ability in a number of educational tracks by the age of twelve. It does not have a national curriculum and allows for wide-ranging autonomy to schools and teachers (OECD, 2014, 2016a). The high level of decentralization is balanced by a strong school inspection mechanism and a national examination system at all levels. The majority of teenagers therefore obtain at least the basic skills in reading, mathematics and science and social sciences as these subjects are an important part of the curriculum. International comparisons show that students attending Dutch schools perform above average, in as well primary as secondary education, comparable to other high performing European and Asian educational systems (Mullis et al., 2016, 2017; OECD, 2016b). The teaching profession does not have an above average status and is seen as underpaid, however the quality of teachers is generally high with the large majority showing good basic teaching skills (OECD, 2016b).

#### South Korea

High academic achievement is greatly prized in South Korea and tracking starts at the age of fourteen, which is the same as the OECD average (OECD, 2016a). One of the major learning resources is government endorsed textbooks and ICT (Heo et al., 2018). The South Korean system greatly emphasizes teaching quality and ongoing development in the teaching profession. It is among the top performing educational systems showing excellent performance in PISA and TIMSS (Mullis et al., 2016, 2017; OECD, 2016b). South Korea’s performance reveals a low percentage of underachieving students, and high percentages of excellent students.

Teachers are recruited from the top graduates, with strong financial and social incentives: high social recognition as well as opportunities for career advancement and beneficial occupational conditions (Kang and Hong, 2008; OECD, 2016a; Heo et al., 2018). In general, education in South Korea is more teacher centered than in other countries, although since 2003 new policies regarding the “7th National Curriculum” have been implemented to focus more on students and student autonomy (Kim, 2003).

#### South Africa

The South African educational system has been functioning poorly at the macro level. Comparative studies show that South African students have very low literacy and numeracy levels, and it has also been ranked last in TIMMS 2015 for mathematics and sciences (Mullis et al., 2016). The overall quality of education has also been ranked as poor (Baller et al., 2016). Reasons for this poor performance might be students instructed in a second language (English), lacking socio-economic resources of students, the legacy of apartheid education and poorly qualified teachers. However, after the apartheid education system, a period of rapid democratization and transformation followed. Changes were evident in curricula that strived to ensure access to education for previously disadvantaged students and to accommodate diverse cultures. Now approximately 15% of the government budget is spent on education.

However, teachers experience a lack of reading resources (Zimmerman and Smit, 2014) and a majority of teachers feels unprepared and inadequately trained for differentiated learning activities (Lomofsky and Lazarus, 2001; Holz and Lessing, 2002). Two other issues that impede inclusive education could be insufficient teacher training in effective teaching such as differentiated instruction (Dalton et al., 2012) and students’ inadequate English proficiency skills (Neeta and Klu, 2013). With 11 official languages, students are instructed in a second language, namely English (Spaull, 2013), which contributes to students’ unclear interpretations of concepts and low performance in major subjects. Low levels of competence in English as instruction language and not being instructed in their home language, impede South African students’ academic performance (Cheatham et al., 2014). In the sample all cultures participated, but mostly students with low socio-economic status.

#### Indonesia

In the Indonesian educational law it has been stated that all citizens have the right to high quality education. The central and local governments therefore provide funds to support free basic education. Despite the diversity with different cultures, religions, ethnics and languages, Indonesia is united in prioritizing education. The average education spending increases significantly each year. In 2017 the World Bank showed that Indonesia education spending is 20.6% (Fasih et al., 2018).

Based on TIMSS and PISA, Indonesia has been consistently ranked amongst the lowest performing educational systems (Mullis et al., 2016). There are many factors that contribute to the low quality of education in Indonesia, including the quality of teachers. Although teachers should take a certification program to improve their teaching, it does not require the teachers to implement or demonstrate their knowledge and skills in the classroom (de Ree, 2016). Most teachers employ a teacher-centered approach instead of student-centered approaches. Other issues including teacher motivation, teacher selection, and initial teacher training programs are mentioned as factors explaining the low quality of education in Indonesia (de Ree, 2016; Fasih et al., 2018).

#### Spain

Spain performs around the average on PISA and TIMMS, but regional differences are relatively large (Hippe et al., 2018). These large differences are assumed to be due to the decentralized government model in which the central government does not advocate all the competences in education (Martínez-Usarralde, 2015). The Southern region scores just above 470 points on PISA, whereas the capital of Madrid and the North-West score above 500 and closer to the Dutch average performance. Teacher training for primary education takes 4 years and is completed with a university degree (*Grado en Maestro de Educación Infantil o Primaria).* Teacher training for secondary education requires a relevant university degree (*Grado*) and an additional master in Teacher Training (Master’s Degree in Teacher Training in Secondary and Upper Secondary Education and Vocational Training) (EURYDICE, 2020).

#### Turkey

The Ministry of National Education (MEB) is responsible for the educational administration under a national curriculum in Turkey. The third level, compulsory secondary education is a 4-year (15–19 age) educational process that prepares students at general, vocational, and technical high schools for the future. In these schools, programs implemented by MEB, set forty class hours in the weekly course schedule that vary depending on the track, curriculum, elective courses in the area and branch. Students are awarded to graduating high school diploma (Ministry of National Education [MEB], 2019a; EURYDICE, 2020). Turkey has a central examination system and is searching more effective and more qualified learning environments in education with some alterations. Over the years Turkey has made significant improvements in education). However, participating in the international testing has revealed a number of educational challenges (e.g., Ministry of National Education [MEB], 2019b) that require patience, hard work, and roadmaps to advance (Ministry of National Education [MEB], 2018). Teacher education programs are determined by the Council of Higher Education (YOK) and carried out at university’s education faculties (Yüksek Öǧrenim Kurumu, the Council of Higher Education [YOK], 2018). The teacher profession has quite high respect and recognition in the Turkish society (Dolton et al., 2018).

The six countries share some similarities and differences in terms of cultural dimensions and educational performance. There are at least three cultural dimensions depicting the diversity and the similarity of the six countries that are relevant to this study: Power Distance index (PDI), Individualism versus Collectivism (IDV), and Indulgence versus Restraints (IVR)^1^ (Hofstede et al., 2010). Of the six countries, Netherlands has the lowest score (PDI = 38). The Dutch society is characterized by being independent, hierarchy for convenience only, and equal rights. Superiors facilitate, empower, and are accessible. Decentralization of power is applied in which superiors count on the experience of their team members. Employees expect to be consulted. Control is disliked, attitude toward superiors are informal, and communication is direct and participative. Spain (PDI = 57), South Korea (PDI = 60), Turkey (PDI = 66) and Indonesia (PDI = 78), respectively have higher power distance scores. In high power distance countries, people are dependent on hierarchy. Superiors are directive and controlling. Centralized power is applied in which obedience to superiors is expected. Communication is indirect and people tend to avoid negative feedback (Hofstede, 2001; Hofstede et al., 2010).

Of the six countries, Netherlands scored the highest in IDV (80), meaning that the country is characterized by a highly individualist society. In this country, a loosely-knit social framework is highly preferred. Individuals are expected to focus on themselves and their immediate families. The superior/inferior relationship is based on mutual advantage, and meritocracy is applied as a base for hiring and promoting individuals. Management focuses on the management of individuals. The remaining countries are considered collectivistic, with Indonesia as the most collectivistic (14), followed by South Korea (18), Turkey (37), and Spain (51), respectively. In the collectivistic society, a strongly defined social framework is highly preferred. Individuals should conform to the society’s ideals and the in-groups loyalty is expected. Superior/inferior relationships are perceived in moral terms like family relationships. Management focuses on management of groups. In some collectivistic countries like Indonesia, there is a strong emphasis on (extended) family relationships, in which younger individuals are expected to respect older people and taking care of parents is highly valued (Hofstede, 2001; Hofstede et al., 2010).

With a score of 68 in IVR, the Dutch society is characterized as being indulgent. This dimension is defined as the extent to which desires and impulses are controlled. The Dutch society generally allows for gratification of desires, being optimistic and enjoying life deliberately. The remaining countries are considered restraint, with South Korea as the most restraint (29), followed by Indonesia (38) and Spain (44). For Turkey with an intermediate score of (49), the characteristic corresponding to this dimension cannot be clearly determined. In restraint cultures, people have a tendency to cynicism and pessimism. In contrast to Indulgent societies, restraint societies do not put much emphasis on leisure time and control the gratification of their desires. People with this orientation have the perception that their actions are restrained by social norms and feel that indulging themselves is somewhat wrong (Hofstede, 2001; Hofstede et al., 2010).

With respect to educational performance, the latest worldwide study of the Programme for International Student Assessment (PISA)^2^ 2018 showed that South Korea’s performance was well above the OECD average and listed among the top 5. Netherlands’ average performance was also above the OECD average but below the South Korean performance. Spain was positioned slightly below the OECD average. Turkey’s mean performance in mathematics improved in 2018 while enrolling many more students in secondary education between 2003 and 2018 without sacrificing the quality of the education provided. Indonesia was listed well-below the OECD average and the lowest compared to the other four countries (OECD, 2019).

## Materials and Methods

### Sample and Procedure

This study was based on a large international project aimed at comparing effective teaching behavior internationally. The project began in Netherlands, with a focus on supporting teacher professional development for novice and experienced teachers. In this study, we included the large student data on teaching behavior in secondary education from six countries: Netherlands (*N*_student_ = 5398), Indonesia (*N_student_* = 4565), South Africa (*N*_student_ = 2678), South Korea (*N*_student_ = 6659), Spain (*N_student_* = 4027), and Turkey (*N_student_* = 6372). Although we aimed at including different types of countries and school systems, within countries the samples are based on generally convenience sampling, which will be elaborated upon in the discussion. Across the countries, data were collected in different years and we used all student-data from Indonesia, South Africa, South Korea and Spain, while focusing on one research year in Netherlands (2015, data are also available for 2014–2018) and Turkey (2017, data are also available for 2018). We made this selection on research years to keep the variability over time as small as possible and to make the sample sizes more comparable across countries. We only included students who have completed all the items on teaching behavior in the student questionnaire. The sample sizes, years of data collection and information on student gender, student age and subjects can be found in Table 1.

**TABLE 1:** Sample sizes and years of data collection.

**Country**	**Student**	**Year of**	**Gender**	**Age**	**Subject**
	**(*N*)**	**observation**	**(%female)**	**(mean**	**(% Maths**
	****	****	****	**and SD)**	**and science)**
Netherlands	5398	2015	52%	14.50 (1.50)	33%
Indonesia	4565	2014	56%	16.30 (0.60)	49%
South Korea	6659	2014	58%	15.40 (1.50)	36%
South Africa	2678	2016	61%	15.30 (1.30)	39%
Spain	4027	2016	49%	15.90 (1.50)	30%
Turkey	6372	2017	56%	16.50 (1.20)	46%
Total	29.669				

In Netherlands, data were gathered across the country. About 85% of the students were in general secondary education and 15% in vocational education. As presented in Table 1, about 33% rated math and science teachers. All schools are public schools. In Indonesia, 85% of the students were in general education and 15% in vocational education. 87% of schools surveyed are public schools. About 76% of the schools are located on Java (the most developed part of the country), and the remaining 24% from Sumatera, Kalimantan, and Sulawesi islands. Most teachers assessed by the students taught math and science subjects (49%), followed by social sciences (30%) and languages (21%). In South Korea, 98% of the students were in general secondary education, and more than half (62%) were in public schools, the other 38% were in private schools. About 80% of the schools are from Chungnam Province, and the remaining 20% from Chungbuk provice. Almost half of the Korean students assessed language teachers (46%), followed by science teachers (36%). In South Africa, only 0.6% of students were in vocational education and 99% of the schools are public schools. Students assessed teachers teaching mathematics and natural sciences (39%), social sciences (37%), followed by 24% languages. Schools are from three provinces: Mpumalanga (52%), Gauteng (24%), and Kwazulu Natal (24%).

In Spain, schools offer general, vocational, and a combination of both: 53.5% of the students were in general education, 0.4% in vocational educational and 45% in a combination of general and vocational education. These students were mostly in public schools (62%). Most students rated language teachers (46%), followed by math and science (30%) and social sciences (28%) teachers. Schools are from three provinces: Asturias (73%), Andalusia (16%), and Galicia (11%). In Turkey, all students were in the general secondary education and in public schools. The largest group of students rated science teachers (43%), followed by languages (36%) and social sciences (21%). Schools are from the highly populated west-north part of the country (Marmara region) that geographically connecting Europe and Asia. Besides its highly social and economic transcontinental contact through history, there is also high internal economical migration to the region which brings and combines the characteristics of other geographical regions and cities of Turkey. In all countries, slightly more female than male students completed the questionnaire. Students were between 11 and 22 years of age.

### Measure

To measure student perceptions of teaching quality, the My Teacher Questionnaire (MTQ) (Maulana and Helms-Lorenz, 2016) was used. This questionnaire was developed based on the observable teaching behavior framework. It consists of 41 items that could be scored on a 4-point Likert scale ranging from 1 (*Completely disagree*) to 4 (*Completely agree*) (see Table 2 for sample items). The questionnaire was translated from English into the target language by a team in each country, and then back-translated in accordance with the guidelines of the International Test Commission (Hambleton, 1994). However, in South Africa the questionnaires were not translated and completed by students in their second language, English. In each country, the translation-back-translation procedure involved an expert team consisting of educational practitioners and a university researcher who were highly knowledgeable about the questionnaire and the theoretical framework underlying the questionnaire. In addition, the expert team are proficient in both English and the target local language. In an earlier research, the 41-items MTQ was proven to be reliable and valid (Inda-Caro et al., 2018).

**TABLE 2:** Factor loadings and total variance explained of the factors in Explanatory Factor Analysis (EFA).

											**Variance explained**
**Netherlands (*N*_total_ = 5398)**											
Learning Climate (5 items)	0.791	0.682	0.762	0.730	0.706						54.1%
Classroom Management (8 items)	0.743	0.662	0.751	0.664	0.694	0.654	0.631	0.757			48.5%
Clarity of Instruction (7 items)	0.584	0.772	0.680	0.763	0.714	0.745	0.693				50.4%
Activating Teaching (10 items)	0.654	0.562	0.732	0.696	0.648	0.737	0.710	0.728	0.811	0.786	50.3%
Differentiation (4 items)	0.777	0.809	0.750	0.782							60.8%
Learning Strategies (7 items)	0.551	0.752	0.722	0.713	0.747	0.719	0.669				48.9%
**Indonesia (*N*_total_ = 4565)**											
Learning Climate (5 items)	0.748	0.597	0.717	0.677	0.644						46.1%
Classroom Management (8 items)	0.658	0.666	0.616	0.626	0.706	0.536	0.476	0.708			39.5%
Clarity of Instruction (7 items)	0.628	0.736	0.585	0.731	0.569	0.574	0.695				42.1%
Activating Teaching (10 items)	0.528	0.472	0.622	0.618	0.562	0.610	0.745	0.646	0.719	0.648	38.7%
Differentiation (4 items)	0.598	0.698	0.755	0.718							48.3%
Learning Strategies (7 items)	0.494	0.682	0.63	0.652	0.657	0.739	0.647				41.5%
**South Korea (*N*_total_ = 6659)**											
Learning Climate (5 items)	0.824	0.740	0.840	0.827	0.789						64.7%
Classroom Management (8 items)	0.765	0.720	0.785	0.740	0.769	0.757	0.782	0.809			58.7%
Clarity of Instruction (7 items)	0.729	0.805	0.766	0.813	0.770	0.754	0.766				59.6%
Activating Teaching (10 items)	0.745	0.728	0.642	0.816	0.794	0.747	0.801	0.796	0.807	0.797	59.1%
Differentiation (4 items)	0.789	0.797	0.824	0.772							63.2%
Learning Strategies (7 items)	0.725	0.817	0.821	0.752	0.789	0.785	0.752				60.5%
**South Africa (*N*_total_ = 2678)**											
Learning Climate (5 items)	0.776	0.734	0.780	0.757	0.706						56.4%
Classroom Management (8 items)	0.710	0.717	0.716	0.710	0.753	0.690	0.694	0.724			51.1%
Clarity of Instruction (7 items)	0.699	0.790	0.766	0.768	0.689	0.746	0.682				54.1%
Activating Teaching (10 items)	0.687	0.677	0.651	0.731	0.718	0.728	0.748	0.728	0.706	0.720	50.4%
Differentiation (4 items)	0.713	0.744	0.787	0.763							56.6%
Learning Strategies (7 items)	0.659	0.719	0.713	0.738	0.736	0.719	0.698				50.7%
**Spain (*N*_total_ = 4027)**											
Learning Climate (5 items)	0.749	0.654	0.725	0.674	0.462						43.7%
Classroom Management (8 items)	0.649	0.577	0.558	0.607	0.673	0.605	0.528	0.718			38.1%
Clarity of Instruction (7 items)	0.473	0.627	0.557	0.647	0.573	0.626	0.661				35.8%
Activating Teaching (10 items)	0.499	0.423	0.524	0.668	0.572	0.567	0.723	0.560	0.714	0.671	35.9%
Differentiation (4 items)	0.684	0.647	0.711	0.644							45.1%
Learning Strategies (7 items)	0.307	0.668	0.639	0.622	0.713	0.578					36.5%
**Turkey (*N*_total_ = 6372)**											
Learning Climate (5 items)	0.793	0.749	0.623	0.777	0.725						54.1%
Classroom Management (8 items)	0.776	0.639	0.690	0.580	0.786	0.652	0.726	0.793			50.2%
Clarity of Instruction (7 items)	0.673	0.801	0.789	0.794	0.717	0.806	0.785				59.0%
Activating Teaching (10 items)	0.702	0.662	0.139	0.814	0.722	0.761	0.827	0.741	0.817	0.816	52.8%
Differentiation (4 items)	0.773	0.780	0.833	0.818							64.2%
Learning Strategies (7 items)	0.686	0.796	0.797	0.744	0.805	0.763	0.731				58.0%

### Analytic Approach

We started with exploratory factor analyses (EFA) using a continuous approach to show the factor structure in each country and estimated reliability scores for each teaching behavior domain in each country. Next, we tested the fit of the model in each country separately using confirmatory factor analyses (CFA). After the measurement model in each country was confirmed, multi-group confirmatory factor analysis (MGCFA) combining all country data was performed. All analyses were done using MPlus version 8.1 (Muthén and Muthén, 2019). Three levels of measurement invariance were tested, respectively.

First, configural measurement invariance tests whether the same factor structure of perceived teaching behavior can be applied on the scores in each country (in all countries all items load on the same factor). This means that instead of letting the statistics decide which items fit together, we imposed our theoretical model on the data. Furthermore, we restricted this factor model to be the same in each country. Second, metric invariance tests whether factor loadings are equal across countries. When the model has an acceptable fit, this means that the relationship between the items and the latent constructs is more or less of the same size in each country. When we obtain metric invariance it becomes possible to assess relationships between latent variables and exogenous factors in the model. Third, scalar measurement invariance tests whether, besides factor structure and factor loadings, the intercepts of the items are equal across countries. Establishing scalar invariance means that we can meaningfully compare the means (μ) of the factors (i.e., teaching domain) across countries (Byrne, 2013).

The common goodness of fit indices for categorical CFA and MGCFA models with an WRMR estimator include the root mean square error of approximation (RMSEA), the comparative fit index (CFI), and the Tucker-Lewis index (TLI), and adhere to common guidelines (i.e., RMSEA < 0.08; CFI > 0.90; TLI > 0.90, also for larger groups RMSEA < 0.07 and SRMR < 0.09 are used) for an acceptable model fit (Hu and Bentler, 1999). A second approach to assess the measurement invariance is to test the deterioration of the model fit between the configural, metric, and scalar model. Changes in CFI (ΔCFI), TLI (ΔTLI) and RMSEA (ΔRMSEA) of <0.01 are deemed acceptable (Cheung and Rensvold, 2002). For relatively large sample sizes, a more liberal ΔCFI value of 0.02 and ΔRMSEA value of 0.03 is to evaluate metric invariance (Rutkowski and Svetina, 2014).

## Results

To what extent do student perceptions of teaching behavior have an invariant internal structure?

### Exploratory Factor Analyses and Reliability Analyses

Preliminary exploratory factor analysis (EFA) results for each country show that items load on the latent factors as intended (see Table 2), indicating that configural measurement invariance (the items load on the same factors in each country) might be evident in the confirmatory factor analysis (CFA). Results of reliability analyses (Cronbach’s alpha) show that all teaching behavior domains have sufficient reliability (see Table 3). However, the reliability of the differentiation domain in Indonesia (Cronbach’s α = 0.64) and that of learning climate in Spain (Cronbach’s α = 0.64) are below the traditional cut-off of 0.70. In addition, McDonald’s omega, which is a more appropriate indication of reliability for ordered categorical variables such as the MTQ, showed generally higher coefficients for the MTQ domains compared to Cronbach’s alpha. The omega coefficient for differentiation domain in Indonesia is exactly within the cut-off (ω = 0.70), and the omega coefficient for learning climate in Spain is close to the cut-off (ω = 0.68). Nevertheless, the omega coefficient for differentiation domain in Spain is still relatively low (ω = 0.60) (see Table 2). The question is, if this remains a problem in confirmatory factor analysis. Nevertheless, low reliability according to Cronbach’s α does not (have to) affect the “true” internal consistency of the scores as assessed in the confirmatory factor analysis framework. Furthermore, one of the reasons to switch from an EFA and Cronbach’s alpha to CFA is because the former received criticism in recent years for not reliably evaluating internal consistency.

**TABLE 3:** Example of items and reliability analysis (Cronbach’s α and McDonald’s ω) on the six subscales based on student data.

**Sample items per domain (α/ω)**	**Learning climate**	**Classroom management**	**Clarity of instruction**	**Activating teaching**	**Differentiation**	**Learning strategies**
My teacher…	..treats me with respect. ..answers my question	..applies clear rules. ..ensures that I pay attention.	..prepares his/her lesson well. ..explains the purpose of the lesson.	..involves me in the lesson. ..motivates me.	..connects to what I know or am capable of. ..knows what I have difficulty with.	..explains how I need to do things. ..stimulates my thinking.
**All countries (α/ω)**	0.782/0.793	0.854/0.856	0.851/0.854	0.884/0.891	0.770/0.782	0.848/0.854
Netherlands	0.781/0.803	0.843/0.850	0.831/0.831	0.888/0.889	0.783/0.796	0.825/0.825
Indonesia	0.703/0.756	0.778/0.774	0.765/0.774	0.820/0.831	*0.642*/0.695	0.763/0.804
South Korea	0.863/0.864	0.899/0.900	0.886/0.887	0.922/0.923	0.798/0.807	0.887/0.891
South Africa	0.806/0.807	0.863/0.863	0.856/0.859	0.890/0.891	0.742/0.745	0.837/0.838
Spain	*0.641*/0.677	0.758/0.767	0.694/0.699	0.797/0.799	*0.591*/*0.596*	0.706/0.704
Turkey	0.776/0.789	0.855/0.858	0.882/0.884	0.883/0.896	0.813/0.815	0.878/0.879

*Reliability value lower than the common cut-off of 0.70.*

### Confirmatory Factor Analysis (CFA) in Each Country

Running CFAs for each country shows the following results. RMSEA values (0.052–0.072), in combination with SRMR values (0.045–0.055), indicate acceptable fit in all countries, but CFI (0.762–0.884) and TLI (0.744–0.876) values indicate insufficient fit (see Table 4).

**TABLE 4:** Confirmatory Factor Analysis (CFA) on the full theoretical model.

	**RMSEA (90% CI)**	**CFI**	**TLI**	**SRMR**
Netherlands	0.058 [0.057;0.059]	0.870	0.861	0.050
Indonesia	0.070 [0.069;0.071]	0.762	0.744	0.055
South Korea	0.068 [0.067;0.069]	0.884	0.876	0.042
South Africa	0.072 [0.071;0.074]	0.833	0.821	0.048
Spain	0.052 [0.051;0.053]	0.818	0.805	0.046
Turkey	0.065 [0.064;0.065]	0.878	0.869	0.045
				

To improve the model-data fit, we inspected the modification indices for all countries separately. We based a selected model on the Dutch data, since this is the source language of the MTQ. Further estimations indicate that deleting item 10 (“*The teacher explains how I need to do things*.”) and item 30 (“*The teacher makes me feel self-confident with difficult tasks*.”) increased the fit the most in Netherlands and increased the fit (and at least did not deteriorate the fit) in the other countries as well. These two items are apparently not distinctive enough and load on multiple domains of teaching quality (cross-loading). Furthermore, we introduced three correlated errors in the domains *learning climate*, *clarity of instructions*, and between *clarity of instruction* and *activating teaching*. These strategies together increased the fit considerably in all countries (see Table 5, including correlated errors). Although the CFI is low in three countries, RMSEA and SRMR are sufficient enough to consider these results to provide a good starting point for the subsequent multi-group confirmatory factor analyses.

**TABLE 5:** Confirmatory Factor Analysis (CFA) on the selected theoretical model (including correlated errors).

	**RMSEA (90% CI)**	**CFI**	**TLI**	**SRMR**
Netherlands	0.049 [0.048;0.050]	0.920	0.912	0.038
Indonesia	0.068 [0.067;0.069]	0.802	0.783	0.050
South Korea	0.066 [0.065;0.066]	0.904	0.896	0.044
South Africa	0.072 [0.071;0.074]	0.852	0.838	0.044
Spain	0.041 [0.040;0.042]	0.897	0.887	0.044
Turkey	0.066 [0.065;0.066]	0.900	0.891	0.044

### Multi-Group Confirmatory Factor Analysis (MGCFA) Across Countries

In the last step we restricted the (selected) model to be the same in all countries to see if we can make comparisons between countries (see Table 6). We estimated the configural equivalent model first in which we only imposed the same factor structure on the scores in each country, which means that we used the same items in each country and let these items load on the same six latent structures. We found a sufficiently good fitting model, especially when we use the RMSEA and SRMR values combination rule from Hu and Bentler (1999). However, the CFI and TLI values are very close to the 0.90 threshold.

**TABLE 6:** Multi-Group Confirmatory Factor Analysis (MGCFA) for six countries.

	**RMSEA**	**SRMR**	**CFI**	**TLI**	**Model compare**	**ΔRMSEA**	**ΔSRMR**	**ΔCFI**	**ΔTLI**	**Decision**
M1: Configural invariance	0.060 (0.060–0.061)	0.045	0.888	0.878						
M2: Metric invariance	0.061 (0.061–0.062)	0.062	0.880	0.874	M1	0.001	0.017	0.008	0.004	Accept (Δ < 0.01)
M3: Scalar invariance	0.068 (0.068–0.069)	0.075	0.845	0.844	M2	0.007	0.013	0.035	0.030	Partly accept (Δ < 0.03)

In the next step, we imposed the factor loadings to be the same across countries (see Table 5). Netherlands was used as the reference country in the model, so we stated that each country should have the same factor loadings as Netherlands. This decreased the fit, as expected, but only minimally with an RMSEA of 0.061 and SRMR of 0.062, which are both still above the threshold and also the changes in all fit indices is smaller than 0.01 for all fit indices except the ΔSRMR value.

In the last step, we estimated the full scalar invariant model (see Table 6). If this model fits, this means we can make meaningful comparisons between the latent means in the countries on the six domains of teaching behavior. Results show that although the RMSEA value (0.068) and the SRMR value (0.075) still show good fit, the CFI and TLI values have dropped quite a bit. This means that according to Hu and Bentler (1999), comparing latent means across countries can be justified. However, according to Cheung and Rensvold (2002), interpreting comparability of scores at the scalar level should be with cautions. The ΔCFI and ΔTLI values are relatively close to a more liberal cut-off proposed by Rutkowski and Svetina (2014). Because the decrease in fit is still very small for the two fit statistics that are most appropriate and robust (RMSEA and SRMR), comparing latent means of the six teaching behavior domains is deemed acceptable. In Table 7, the standardized factor loadings for each country based on the scalar invariance model are presented.

**TABLE 7:** Standardized Factor Loadings for each country in the scalar invariance model.

**Netherlands (*N*_total_ = 5398)**									
Learning Climate (4 items)	0.561	0.634	0.520	0.704					
Classroom Management (7 items)	0.696	0.565	0.722	0.563	0.690	0.539	0.693		
Clarity of Instruction (7 items)	0.535	0.727	0.634	0.647	0.680	0.673	0.608		
Activating Teaching (9 items)	0.612	0.575	0.622	0.679	0.627	0.686	0.687	0.728	0.748
Differentiation (4 items)	0.665	0.704	0.697	0.692					
Learning Strategies (5 items)	0.684	0.647	0.628	0.630	0.646				
**Indonesia (*N*_total_ = 4565)**									
Learning Climate (4 items)	0.548	0.533	0.482	0.610					
Classroom Management (7 items)	0.581	0.543	0.612	0.508	0.619	0.537	0.618		
Clarity of Instruction (7 items)	0.502	0.617	0.552	0.576	0.543	0.551	0.580		
Activating Teaching (9 items)	0.459	0.498	0.479	0.608	0.556	0.527	0.623	0.619	0.606
Differentiation (4 items)	0.543	0.509	0.568	0.554					
Learning Strategies (5 items)	0.626	0.528	0.575	0.557	0.607				
**South Korea (*N*_total_ = 6659)**									
Learning Climate (4 items)	0.721	0.730	0.713	0.749					
Classroom Management (7 items)	0.742	0.671	0.773	0.649	0.737	0.697	0.767		
Clarity of Instruction (7 items)	0.650	0.742	0.705	0.719	0.766	0.734	0.713		
Activating Teaching (9 items)	0.670	0.698	0.617	0.799	0.745	0.726	0.768	0.780	0.772
Differentiation (4 items)	0.729	0.720	0.744	0.655					
Learning Strategies (5 items)	0.770	0.751	0.760	0.689	0.696				
**South Africa (*N*_total_ = 2678)**									
Learning Climate (4 items)	0.638	0.668	0.640	0.712					
Classroom Management (7 items)	0.661	0.631	0.714	0.598	0.678	0.641	0.695		
Clarity of Instruction (7 items)	0.628	0.725	0.688	0.666	0.662	0.702	0.662		
Activating Teaching (9 items)	0.609	0.597	0.601	0.689	0.667	0.673	0.695	0.696	0.715
Differentiation (4 items)	0.600	0.644	0.685	0.646					
Learning Strategies (5 items)	0.677	0.645	0.649	0.620	0.657				
**Spain (*N*_total_ = 4027)**									
Learning Climate (4 items)	0.515	0.579	0.490	0.655					
Classroom Management (7 items)	0.612	0.490	0.513	0.511	0.590	0.509	0.634		
Clarity of Instruction (7 items)	0.386	0.488	0.502	0.462	0.535	0.521	0.501		
Activating Teaching (9 items)	0.495	0.444	0.495	0.571	0.541	0.569	0.574	0.576	0.618
Differentiation (4 items)	0.501	0.509	0.560	0.494					
Learning Strategies (5 items)	0.538	0.562	0.552	0.530	0.530				
**Turkey (*N*_total_ = 6372)**									
Learning Climate (4 items)	0.648	0.707	0.508	0.730					
Classroom Management (7 items)	0.722	0.597	0.599	0.574	0.724	0.633	0.717		
Clarity of Instruction (7 items)	0.643	0.759	0.735	0.718	0.684	0.759	0.736		
Activating Teaching (9 items)	0.668	0.648	0.447	0.756	0.682	0.699	0.774	0.797	0.768
Differentiation (4 items)	0.707	0.713	0.734	0.706					
Learning Strategies (5 items)	0.744	0.722	0.672	0.688	0.706				

### Robustness Check

Due to the hierarchical structure of the data, we performed a robustness check to ascertain the extent to which the results are valid when the multilevel structure is not taken into account. When the hierarchical structure is ignored, this can lead to analytical and interpretation difficulties (Heck and Thomas, 2015), because the assumptions of (1) independent observations and (2) independent, normally distributed, and heteroscedastic random errors are most probably violated (Kreft and de Leeuw, 1998). Subsequently, we performed multilevel CFA to analyze *within* as well as *between* the levels of the factor structure.

In the current data, students are nested within teachers, teachers are nested within schools, and schools are nested within countries. Due to insufficient sample size at the country level, we chose to take schools as the higher level (level 2) and students as the lower level (level 1) in the first analysis, because we expected that there would be more heterogeneity between schools than within schools that we should control for when our variable of interest is teaching behavior. The multilevel CFA structure thus allows to control for clustering of observations within schools. We performed multilevel models at the country level as well as with all countries. However, the estimated models did not converge if we used normal estimation models. By applying the MUML estimation procedure, we found the same results as with our normal MGCFA analysis presented earlier. This indicates that taking into account the multilevel structure in the model does not affect the outcomes of our analysis.

### How Does Perceived Teaching Behavior Differ Across Countries?

#### Which Countries Were Rated Higher and on Which Teaching Domains?

The latent means based on the full scalar invariant model of scores shows between country variations in the perceived teaching behavior (see Table 8). The order of teaching behavior domains from low to high in the six countries is visible (see Table 9). Perceived learning climate was highest in Netherlands and Turkey, followed by South Korea, South Africa, and Spain. This domain was perceived the lowest in Indonesia. The mean difference between Netherlands and Turkey is not significant (*p* > 0.05). In general, South Korean students scored their teachers highest on the remaining five teaching behavior domains. Dutch teachers were rated second highest for classroom management and clarity of instruction. However, they were rated the lowest for differentiation and teaching learning strategies.

**TABLE 8:** Comparison of latent means of the scalar invariance Multi-Group Confirmatory Factor Analysis (MGCFA) model.

	**Netherlands^1^**	**Indonesia**	**South**	**South**	**Spain**	**Turkey**
	****	****	**Korea**	**Africa**		
CLM^2^	0.000	−1.361***	−0.155***	−0.410***	−0.588***	0.000
ORG	0.000	−1.091***	0.043*	−0.368***	−0.665***	−0.197***
CLR	0.000	−0.660***	0.443***	−0.096***	−0.146***	−0.036*
ACT	0.000	−0.157***	0.642***	0.217***	0.100***	−0.075***
DIF	0.000	0.144***	1.004***	0.396***	0.649***	0.375***
TLS	0.000	1.022***	1.409***	0.870***	0.486***	0.215***

^1^Netherlands is the reference category, *p < 0.05, **p < 0.01, ***p < 0.001. ^2^CLM = Learning Climate, ORG = Classroom Management, CLR = Clarity of Instruction, ACT = Activating Teaching, DIF = Differentiation, TLS = Learning Strategies.

**TABLE 9:** Ranking of latent means of the scalar invariance Multi-Group Confirmatory Factor Analysis (MGCFA) model (including significances).

	**Lowest**					**Highest**
CLM	Indonesia	Spain	South Africa	South Korea	*Turkey*	*Netherlands*
ORG	Indonesia	*Spain*	*South Africa*	Turkey	Netherlands	South Korea
CLR	Indonesia	*Spain*	*South Africa*	Turkey	Netherlands	South Korea
ACT	*Indonesia*	*Turkey*	Netherlands	Spain	South Africa	South Korea
DIF	Netherlands	Indonesia	*Turkey*	*South Africa*	*Spain*	South Korea
TLS	Netherlands	Turkey	Spain	South Africa	Indonesia	South Korea

*Countries in italics do not significantly differ from each other on the latent means (*p* < 0.05). **CLM = Learning Climate, ORG = Classroom Management, CLR = Clarity of Instruction, ACT = Activating Teaching. DIF = Differentiation, TLS = Learning Strategies. **CLM = Learning Climate, ORG = Classroom Management, CLR = Clarity of Instruction, ACT = Activating Teaching, DIF = Differentiation, TLS = Learning Strategies.

Turkish students rated their teachers higher on learning climates (comparable to Netherlands) and classroom management especially when compared with Spain, South Africa, and Indonesia, but they scored their teachers relatively lower in the remaining domains. South African students scored their teachers relatively higher on activating teaching, differentiation, and teaching learning strategies compared to other countries. However, they scored lower on learning climate, clarity of instruction, and classroom management than Turkey, Netherlands, and South Korea. Spanish students scored their teachers higher on differentiation compared to students in South Africa, Turkey, Indonesia, and Netherlands. They also rated their teachers higher on activating teaching compared to students in Netherlands, Turkey, and Indonesia. Finally, Indonesian students rated their teachers the lowest on learning climate, classroom management and clarity of instruction. However, they rated their teachers higher on teaching learning strategies compared to Netherlands, Turkey, Spain, and South Africa.

#### What Is the Most Complex Teaching Behavior Domain Based on Student Perceptions?

**FIGURE 1:**
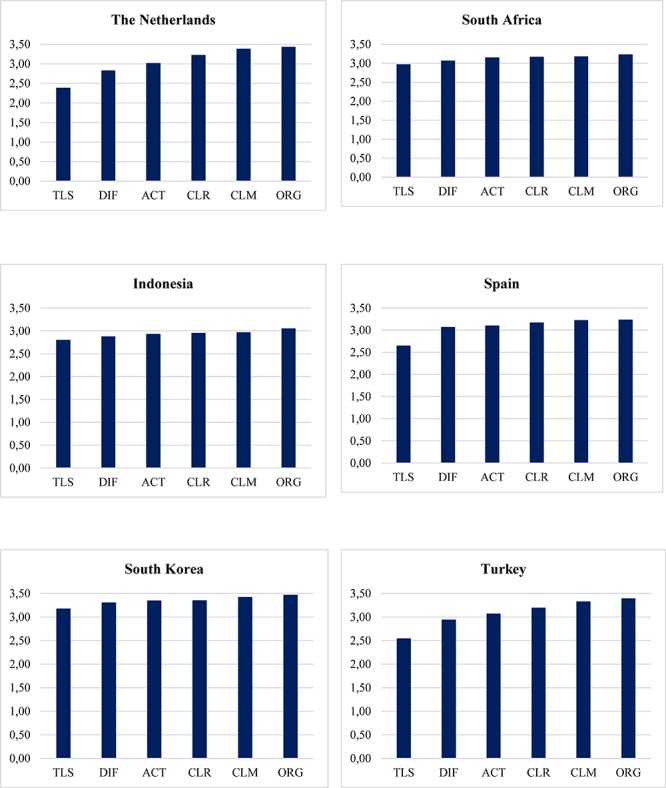
Mean (raw scores) of teaching behavior domains across countries. **CLM = Learning Climate, ORG = Classroom Management, CLR = Clarity of Instruction, ACT = Activating Teaching, DIF = Differentiation, TLS = Learning Strategies.

As indicated earlier, the measurement model of the six teaching behavior domains is confirmed in the six countries. Based on the raw mean scores of teaching behavior domains across countries, we found an interesting general pattern (see Figure 1). According to Maulana et al. (2015a), the mean scores can be interpreted qualitatively based on the original measurement metric as follows: 1.00–2.00 (low/insufficient), 2.01–3.00 (moderate/sufficient), and 3.01–4.00 (high/good).

In all six countries, teaching learning strategies were generally rated the lowest. Specifically, this teaching domain was rated the lowest in Netherlands (*M*_Netherlands_ = 2.39, *SD* = *0.71*), followed by Turkey (*M_Turkey_* = 2.55, *SD* = *0*.85), Spain (*M*_Spain_ = 2.65, *SD* = *0*.66), Indonesia (*M*_Indonesia_ = 2.81, *SD* = *0*.49), South Africa (*M*_South Africa_ = 2.97, *SD* = *0*.75), and South Korea (*M*_South Korea_ = 3.18, *SD* = *0*.61). On average, perceived teaching learning strategies in South Korea was perceived as high, while in the remaining countries it was perceived as moderate.

Furthermore, differentiation was rated the second lowest in Netherlands (*M*_Netherlands_ = 2.83, *SD* = *0.*67), Indonesia (*M*_Indonesia_ = 2.88, *SD* = *0*.46), South Africa (*M*_South Africa_ = 3.07, *SD* = *0*.71), and South Korea (*M*_South Korea_ = 3.31, *SD* = 0.54). On average, students perceived differentiation in Indonesia and Netherlands as moderate, while in South Africa and South Korea as high. In Spain and Turkey, differentiation was rated relatively higher (*M*_Spain_ = 3.10, *SD* = *0*.53 *M*_Turkey_ = 3.07, *SD* = 0.74) than activating teaching (*M*_Spain_ = 3.07, *SD* = 0.49 *M*_Turkey_ = 2.94, *SD* = 0.69), placing activating teaching as the second lowest in the two countries. On average, differentiation was perceived as high/good in Spain and Turkey. Unlike in the other four countries, learning climate in Indonesia (M_Indonesia_ = 2.92, *SD* = 0.47) and South Korea (M_South Korea_ = 3.35, *SD* = 0.51) was rated as relatively more complex, albeit at the sufficient (Indonesia) and good (South Korea) level.

## Discussion

Teachers’ teaching behavior is strongly related to students’ learning outcomes (Seidel and Shavelson, 2007; Hattie, 2009), but how teaching behavior is perceived by students across countries is relatively unclear. Because what students will learn in the classroom depends on how they perceive, interpret, and process the information during teaching practices (Shuell, 1996), insights regarding student perceptions of teaching behavior from various cultural contexts can contribute to the advancement of knowledge of effective teaching behavior. The novel contribution of the current study is that we investigated measurement invariance of perceived teaching behavior across six cultural contexts including Netherlands, Spain, Turkey, South Africa, South Korea, and Indonesia. Furthermore, the study attempted to compare perceived teaching behavior across countries based on a uniform student measure.

### Reliability and Measurement Invariance of Perceived Teaching Behavior

In terms of domain internal consistencies (Cronbach’s alpha and McDonald’s omega), the six domains of teaching behavior are adequately reliable. However, the reliability of differentiation domain in Spain (α = 0.59, ω = 0.60) is below the conventional cut-off of 0.70 (DeVellis, 2012). Cronbach’s α coefficient is known to be quite sensitive to the number of items in the scale (Pallant, 2016). In the MTQ, differentiation was measured using only four items, and learning climate using five items, which are relatively limited to form high internal consistency. Due to the lengthy form of the MTQ (41 items), it is not wise to add extra items to avoid missing responses and response fatigue which can cause bias in the survey (Rolstand et al., 2011). Nevertheless, the reliability value is still within the acceptable threshold (Murphy and Davidshofer, 2004). McDonald’s omega, which is a more appropriate indication of reliability for ordered categorical variables such as the MTQ, showed generally higher coefficients for the MTQ domains compared to Cronbach’s alpha. Nevertheless, the omega coefficient for differentiation domain in Spain is still relatively low (ω = 0.60). It is likely that the limited number of items of this domain explains the low alpha and omega values. This general tendency is evident that compared other domains, the reliability coefficients of differentiation in the six countries (except in Turkey) are lower.

The issue of reliability is related to the source of variations. Ideally, rating scales should reflect solely the amount of variability in the trait/construct itself. However, variations can also reflect respondents bias or error, or reflect trait-respondent interaction (Rohner and Katz, 1970). In cross-country studies, the interplay between the source of variance components may differ depending on the cultural background (e.g., the tendency of respondents in certain cultures to respond to particular traits in a certain way) and specific context conditions (e.g., survey time, methods of surveys). Because internal consistency of a measure can be influenced by between culture and within culture differences (Moschis et al., 2011), any source of variations in both cultural levels should ideally be taken into account. In practice, it is highly difficult to control cultural factors. Even if one tries to control the two aspects very strictly, there is no guarantee that the undesired source of variations can be reduced significantly due to some complex culture mechanisms that should be investigated in more depth qualitatively.

By applying the MGCFA approach based on the SEM framework to assess measurement invariance of perceived teaching behavior, we found that the six teaching behavior domains show sufficient invariance in the six countries. This allows us to interpret and compare mean scores across the six countries in a meaningful and valid way. This finding is in line with a recent study on student perceptions of teachers’ instructional quality showing sufficient invariance of teacher support, cognitive activation, and classroom management in Australia, Canada, and the United States (Scherer et al., 2016). Our study extends the validity of comparing perceived teaching behavior beyond English speaking countries. It should be noted, however, that not all invariance indices are sufficiently high. This means that the scale properties of the MTQ scales across countries will require further improvement in the future. The current study covers particularly the etic aspect of perceived teaching behavior. We recommend to include both etic and emic aspects together in future toward deeper understanding and improving measurement invariance across cultural contexts.

### Differences in Perceived Teaching Behavior Across Countries

Results suggest that learning climate was perceived to be the highest in Netherlands and Turkey, and the lowest in Indonesia. In Netherlands, research on psychosocial classroom climate has a long tradition and is grounded within the teacher-student relationship framework. Specifically, the importance of learning climates for student learning and outcomes has been studied from the interpersonal teacher behavior framework (Wubbels and Brekelmans, 2005). This framework has been integrated in teacher education as well as in in-service teacher professional development across the country (van Tartwijk et al., 2014). In addition, the integration of teaching effectiveness frameworks into some Dutch teacher education programs and teacher professional development has also been done, putting a strong importance of learning climates as a pre-requisite for more effective teaching behavior (Maulana et al., 2017). On the other hand, the relatively low rating of Indonesian teachers on learning climate may also be associated with the still commonly applied student-centered teaching approach (de Ree, 2016; Fasih et al., 2018).

From a more distal perspective, there is a suggestion that schools in Asia are more examination-oriented and teachers are typically viewed as authoritative figures (Khine and Fisher, 2001). The examination-driven classroom culture is assumed to affect the teachers’ teaching styles leading to less supportive learning climates. Subsequently, classroom environments are often perceived to be better in Western compared to non-Western classes (Liem et al., 2008), which seems to be reflected in our study as well. Past research revealed that students in Australia perceived classroom environments more positively than students in Taiwan (Fraser and Aldridge, 1998). Similarly, students reported more positive classroom environments in Australian, New Zealand, and English teacher classes than in Asian teacher classes (Khine and Fisher, 2001).

Dutch teachers were perceived second highest in classroom management and clarity of instruction, after the Korean teachers. This finding might be related to the Dutch educational system, which strongly emphasizes classroom management as one of the first skills that need to be developed by teachers during teacher education. The implementation of realistic teacher education in Netherlands has prioritized classroom management skills to be mastered by novice teachers (van Tartwijk et al., 2011). In addition, efforts to integrate the mastery of classroom management skills using an interpersonal approach has been made (Wubbels et al., 2006), which could promote effective classroom management and improve learning climates simultaneously. However, our study revealed that differentiation and teaching learning strategies were perceived less positively in Netherlands. This finding is consistent with past studies indicating that Dutch teachers are still struggling with the implementation of these two teaching domains in their daily classroom practices (Maulana et al., 2017).

Furthermore, we found that South Korean students perceived their teachers highest on all teaching domains, except on learning climate (third highest after Turkey and Netherlands). It should be noted, however, although the difference in the mean score of learning climates between South Korea and Turkey/Netherlands is statistically significant, the difference is rather small. Given that South Korean teachers are recruited from the top graduates, with strong financial and social incentives as well as high social recognition and promising opportunities for career advancement and beneficial occupational conditions (Kang and Hong, 2008; OECD, 2016b; Heo et al., 2018), it is expected that only highly effective teachers enter the teaching profession in the country, which seems to be reflected from the lens of their students captured by the current study. There is a skepticism, however, that education in South Korea is more teacher-centered than in other countries, although since 2003 new policies regarding the “7th National Curriculum” have been implemented to focus more on students and student autonomy (Kim, 2003). This doubt is not reflected in the current student perceptions.

Turkish students reported relatively higher ratings on learning climates and classroom management especially when compared to Spain, South Africa, and Indonesia. Findings of several studies in the Turkish context are in line with the current study, indicating that Turkish (science) classroom climates were perceived as having high quality by the students (den Brok et al., 2010; Telli, 2016). Interestingly, South African teachers received relatively higher ratings on activating teaching, differentiation, and teaching learning strategies compared to Spain, Turkey, Indonesia, and Netherlands. However, South African students rated their teachers lower on learning climate, classroom management, and clarity of instruction than their colleagues in Turkey, Netherlands, and South Korea. The reason for a high rating in differentiation and low rating in clarity of instruction could both be attached to second language instruction in classes. Teachers need to clarify all concepts and apply to real life situations to improve understanding of abstract concepts. Past studies indicated that the majority of South African teachers felt insufficiently prepared and lack skills for including all students in high quality teaching including differentiation (Holz and Lessing, 2004; de Jager, 2013).

Spanish students rated their teachers higher on differentiation compared to students in South Africa, Turkey, Indonesia, and Netherlands. The reason for this might be related to recent educational acts taking place in the country emphasizing diversity and educational needs for all students as key concepts of the contemporary educational practice. They also rated their teachers higher on activating teaching compared to students in Netherlands, Turkey, and Indonesia. Reasons for this finding remain unclear due to the lack of systematic research on teaching behavior in the country (Fernández-García et al., 2019). The TALIS-PISA link study on teacher perceptions on their teaching behavior showed that Spanish teachers perceived activating teaching rather high as well, but they perceived rather low on teaching learning strategies (OECD, 2016a). Finally, Indonesian students rated their teachers the lowest on learning climate, classroom management and clarity of instruction, which may explain the low performance of Indonesian students in the international testing (Mullis et al., 2016). However, they rated their teachers higher on teaching learning strategies compared to Netherlands, Turkey, Spain and South Africa. Although reasons for this finding remain unclear, this might be related to the ongoing efforts of improving teaching quality in Indonesia, emphasizing the importance of treating students as active learners instead of viewing them as receivers of knowledge (World Bank, 2018).

On average, we found a general tendency that perceived teaching learning strategies were perceived as the lowest in the six countries. This suggests that this teaching domain appears to be the most complex teaching skill for teachers. Differentiation was perceived as the second lowest in all countries, except in Spain and Turkey in which activating learning was rated lower than differentiation. In general, this finding seems to suggest that teaching learning strategies, differentiation, and to some extent activating teaching appear to be perceived as more complex in the six countries compared to learning climates, classroom management, and clarity of instruction, which is in line with previous studies (Pietsch, 2010; van de Grift et al., 2014).

Our finding may suggest that, in general, teachers in the six countries are still dealing with concerns related to the self and tasks, and not so much with concerns related to the impact on their students yet (Fuller, 1969). This might not apply to South Korean teachers who received high ratings in all domains of teaching behavior, including differentiation and teaching learning strategies. This may indicate that South Korean teachers, in general, are already concerned about making impacts on their students. The results may be reflected in the top performance of their students internationally (Mullis et al., 2016, 2017; OECD, 2016b).

Based on the original metric, perceived differentiation is also high in Turkey, Spain, and South Africa. Based on the 2015 PISA data, Turkish teachers showed a great effort to respond to the individual needs of their students (Özkan et al., 2019). Albeit the similarity regarding the complexity level of differentiation and teaching learning in our study using classroom observation, we observed a reverse order of complexity between student perceptions and observer observations, in which students perceived learning strategies as the lowest, while observers rated differentiation as the lowest. Nevertheless, both students and observers agreed generally that teaching learning strategies and differentiation are two teaching domains that seem to be highly complex in the countries. This is consistent with the literature mentioning that teachers often find differentiating instruction challenging to implement in practice (Tomlinson et al., 2003; Subban, 2006). The probability of a teacher to implement differentiation within classrooms increases when other teaching behavior domains are demonstrably better. Differentiation is related to other domains in a stage-like manner in which differentiated instruction is one of the demanding domains of teaching behaviors that is typically seen in the lessons of highly effective teachers who incorporate behaviors from other domains in their lessons too (Pietsch, 2010; Maulana et al., 2019). Teachers with relatively high teaching quality, are more likely to teach in a student-centered manner and take into account student differences into their teaching (Pietsch, 2010).

Finally, it is interesting to note an emerging general pattern with regard to the cultural dimensions of Power Distance, Individualism versus collectivism, and Indulgence versus Restraint (Hofstede, 2001; Hofstede et al., 2010). From the current study the impression rises that students’ perceptions seem to be the most positive in a context of moderate power distance, higher levels (though not extreme high) of collectivism and higher levels of restraint. Cultural contexts with higher levels of indulgence seem to be related to lower student perception scores regarding complex behavioral teaching domains except for Indonesia. Future research is needed to confirm these and other macro-level context factors that might inhibit or facilitate student perceptions of their teachers.

### Implications for Research and Teaching

The international research project underpinning the current study focuses on cross-country comparison of teaching quality. The main goal is to gain insights into teaching practices across countries, which can stimulate cooperation and collaboration to improve teaching quality internationally. The current study confirms the relevance of the generic domains of teaching behavior, as measured by the MTQ initially developed in the Dutch context, in the six contrasting cultural contexts. The study also reveals some similarities and differences in teaching behavior across the six countries, which suggests the importance of etic and emic perspectives to understanding teaching behavior.

South Korean teachers were rated high in the six domains of teaching behavior, including the two most complex domains of differentiation and teaching learning strategies, which is in agreement with the previous studies using classroom observations. It might be that South Korean teachers hold strong values of making impact on their students (concern with student impact) and reflect these values in daily teaching practices more than teachers in other countries. Subsequently, teachers in other countries (especially the ones included in this study) may want to learn from South Korean teachers regarding ways and strategies to improve teaching learning strategies skills that can result in higher student ratings on these two domains particularly, and in all teaching domains generally.

### Limitations and Future Directions

Several limitations should be considered when interpreting results of the present study. First, given that the data was collected based on a mostly convenience sampling approach, generalizations of findings to the country level is limited. We therefore encourage improved sampling designs (e.g., stratified sampling), as for example discussed by Kaminska and Lynn (2016) to address the issue of generalizability, so that more representative descriptions of teaching behavior across countries can be documented. Samples that are more representative for the country will lead to more generalizable results. Second, our sample comprises six countries. This means that findings related to measurement invariance of perceived teaching quality merely apply for these countries. It remains unknown how universal the teaching quality construct is, especially as measured by the MTQ. This is also the case because some of the cut-off values for the MGCFAs were quite low, which means that another avenue of research would be to search for partial scalar invariance when adding more countries. Hence, we recommend larger scale student surveys involving more educational contexts across various cultural backgrounds to test for teaching behavior construct comparability so that a more international teaching quality construct can be established that allows for more global insights in teaching quality.

Third, the reliability value of differentiation in Spain is relatively low. Although differentiation has adequate reliability in the remaining five countries, the values are still smaller compared to other domains having more items. Future research should try to add more items to this domain to improve reliability (Tavakol and Dennick, 2011), and try to employ more advance techniques (e.g., hierarchical IRT) to assess reliability taking into account item and respondent characteristics. Fourth, the current study relied solely on student perceptions. Student and teacher perceptions can be affected by multiple factors (e.g., social desirability, cultural values, gender), which may reduce the objectivity of this technique (Aleamoni, 1981). Particularly, the way students in the six countries responded to the surveys may be affected by how they value power distance, individualism, and indulgence in their cultures (Hofstede et al., 2010). Because MGCFA is a variable-centered approach, future research may benefit from adding a person-centered approach to study measurement invariance and country comparison in perceived teaching behavior. A person-centered approach allows researcher to examine respondent behaviors that can be coupled with their cultural background. Results from self-report studies should be interpreted with care and should not be over extrapolated (Saljo, 1997). Fifth, given the hierarchical structure of the current study, one may argue that multilevel CFA should be applied instead of the general CFA. However, using multilevel analysis on SEM models is relatively new, and SEM software packages are limited in addressing the complexities of multilevel models adequately (Byrne, 2013). Future research should gather sufficient higher level data to allow for multilevel SEM.

Finally, South Korean teachers received high ratings in all domains of teaching behavior, including differentiation and teaching learning strategies. Although this finding may indicate that South Korean teachers, in general, are already concerned about making impacts to their students in their teaching practices from students’ point of view. The conjecture related to South Korean concerns stage and their teaching quality as well as the partial (in)consistency in findings between student perceptions and observer ratings require a more in-depth investigation in future research.

Given that both observations and student surveys have strengths and weaknesses, both methods should be seen as complementary ways to gather information about teaching behavior (triangulation). Triangulation can ensure the validity and reliability of instruments measuring complex classroom practices (Denzin, 1997). However, Riggin (1997) argued that triangulation can result in either complementary or conflicting findings. In the latter case, a more in-depth investigation into the sources of inconsistency and the underlying mechanisms should be done by incorporating sound theories that can provide more understanding about perceptions constructed by individuals given their (cultural) background. Reasoned action approach theory (Fishbein and Ajzen, 2010) and sources of independent variance in perception theory (Kenny, 2004) might be worth considering in future research.

## Data Availability Statement

The datasets generated for this study are available on request to the corresponding author.

## Ethics Statement

The Institutional Review Board (IRB) of the Department of Teacher Education was established in January 2017. Research projects which were started before this official installation of the IRB did not require an approval from the IRB. All research projects before this date were reviewed and approved by the Director of the department. The current study wasstarted at the end of 2014. Although an IRB did not exist yet during that time, studies conducted within the department followed the Netherlands Code of Conduct for Academic Practice (2014) and the Code of Ethics for research in the Social and Behavioral Sciences Involving Human Participants (2016).

## Author Contributions

SA wrote sections of the manuscript and performed statistical analyses. RM conceived and designed the study, wrote sections of the manuscript, checked statistical analyses, and coordinated the manuscript. MH-L contributed to the conception, design, and writing of the study. ST, SC, C-MF-G, TJ, YI, MI-C, OL, RS, TC, and MJ contributed to organizing databases and writing sections of the manuscript. All authors read and approved the submitted version.

## Conflict of Interest

The authors declare that the research was conducted in the absence of any commercial or financial relationships that could be construed as a potential conflict of interest.

## Footnotes

1 The country data for South Africa related to these cultural values is not available. The current available data of South Africa is limited to the White population only, which is a minority group in the country.

2 South Africa did not participate in the PISA study. Hence, the performance data for South Africa is not available.
